# A New Label-Free Technique for Analysing Evaporation Induced Self-Assembly of Viral Nanoparticles Based on Enhanced Dark-Field Optical Imaging

**DOI:** 10.3390/nano8010001

**Published:** 2017-12-22

**Authors:** Ima Ghaeli, Zeinab Hosseinidoust, Hooshiar Zolfagharnasab, Fernando Jorge Monteiro

**Affiliations:** 1 i3S-Instituto de Investigação e Inovação em Saúde, Universidade do Porto, 4200-135 Porto, Portugal; 2 INEB, Instituto de Engenharia Biomédica, 4200-135 Porto, Portugal; 3 Departmento de Enginharia Metalurgia e Materiais, FEUP, Faculdade de Engenharia, Universidade do Porto, 4200-465 Porto, Portugal; 4 Department of Chemical Engineering, McMaster University, Hamilton, ON L8S 4L7, Canada; shdoust@gmail.com; 5 Departamento de Engenharia Eletrotécnica e de Computadores, FEUP, Faculdade de Engenharia, Universidade do Porto, 4200-465 Porto, Portugal; hooshiar.h.z@ieee.org

**Keywords:** nanoparticle self-assembly, T4 phage nanoparticle suspension, enhanced dark-field microscopy, nanoparticle tracking analysis, label-free technique, drying-induced self-assembly

## Abstract

Nanoparticle self-assembly is a complex phenomenon, the control of which is complicated by the lack of appropriate tools and techniques for monitoring the phenomenon with adequate resolution in real-time. In this work, a label-free technique based on dark-field microscopy was developed to investigate the self-assembly of nanoparticles. A bio-nanoparticle with complex shape (T4 bacteriophage) that self-assembles on glass substrates upon drying was developed. The fluid flow regime during the drying process, as well as the final self-assembled structures, were studied using dark-field microscopy, while phage diffusion was analysed by tracking of the phage nanoparticles in the bulk solutions. The concentrations of T4 phage nanoparticles and salt ions were identified as the main parameters influencing the fluid flow, particle motion and, consequently, the resulting self-assembled structure. This work demonstrates the utility of enhanced dark-field microscopy as a label-free technique for the observation of drying-induced self-assembly of bacteriophage T4. This technique provides the ability to track the nano-sized particles in different matrices and serves as a strong tool for monitoring self-assembled structures and bottom-up assembly of nano-sized building blocks in real-time.

## 1. Introduction

Viral nanoparticles (VNPs) can be used as building blocks for developing advanced, functional materials, such as bioactive surfaces, flexible optics and electronics, biosensors, and drug/gene delivery vehicles for biomedical engineering [1,2,3,4].

Various structures, shapes, sizes, and properties of viruses, as well as their amino acid groups, increase their benefits for the potential use as precursors to create inorganic nanostructures. Wang et al. [5], summarized the studies on the self-assembly of different types of viruses to design and fabricate biomimetic nanostructures for sensor applications. VNPs can self-assemble into two- or three-dimensional macrostructures. The patterning of virus-based nanomaterials on a substrate (surface-specific virus assembly) as a requirement for advanced engineering applications, such as nanowires and catalysis, has been reviewed by Lee et al. [6]. The interfacial and biological methods through implementing of biological recognition (selectively immobilized viruses), and mechanical and interfacial forces, have been reported for such patterning. Both substrate properties and surface characteristics of the viruses are influential on the formation of top virus layers onto the solid substrates [6]. Even though specific affinities of viruses to the substrate might be helpful for a targeted patterning, the specific interaction might result in higher binding energies, causing more defects in virus-assembled structures. Additionally, the specific interactions may be limited to one substrate material. Hence, nonspecific virus assembly on solid substrates is a concern [7]. 

Nonspecific assembly of VNPs on solid surfaces can be achieved through methods such as the Langmuir-Blodgett technique, convective assembly, and droplet evaporation [8,9,10,11], thus resulting in the bottom-up assembly of the nanoparticles into two-dimentional films that exhibit various degrees of structure, such as honeycomb, coffee rings, and nanocrystal superlattices [12]. A tunable crack-free ultrathin film with highly-ordered assembly of nanoparticles into supperlattices could be obtained through a controlled evaporation-induced self-assembly procedure [13,14].

Several researchers have investigated the two-dimensional ordered structures formed by droplet evaporation self-assembly of VNPs [3,15,16], mainly focusing on icosahedral or rod-shaped viruses because of their simpler geometry. Fully dehydrated suspensions (if stored in a humidity- and temperature-controlled environment) are static and fairly stable because the self-organization process is complete. At this point, more invasive endpoint methods, such as scanning electron microscopy (SEM) [17,18], X-ray crystallography, and atomic force microscopy (AFM) [19,20] can be safely applied, all of which can result in high resolution endpoint data. Although some of these techniques (namely SEM and AFM) can, in theory, be carried out in the liquid mode, these techniques require meticulous sample preparation, which is invasive and will interfere with the self-organization process [21], making real-time, non-invasive visualization of VNPs (and nanoparticles in general) in a fluid matrix a challenging task. Optical methods, such as light scanning microscopy (LSM) [22], incident light microscopy [23], fluorescence microscopy [17,24], and confocal laser scanning microscopy [25,26,27], have proven useful to various degrees for non-invasive, real-time monitoring of nanoparticle suspensions. Optical methods are by far the simplest and less invasive techniques for this purpose; however, being very small in size, with ultra-weak polarization forces and low dielectric constants, it is difficult to discriminate the nanoparticles from the surrounding medium using optical methods [28]. Fluorescence labelling allows for real-time imaging of nanoparticles with reasonable resolution in a wide range of matrices; however, this method is invasive and is challenged by limitations of the fluorescence emission lifetime and photo-bleaching [29,30,31]. Moreover, the Brownian motion of fluorescent micro- and nanoparticles causes the particles to tumble and blink irregularly, decreasing the signal-to-noise ratio in fluorescence microscopy [32]. 

Recently, several attempts have been made to develop label-free, real-time, sensitive, and low-cost techniques for visualizing VNPs and other nanoparticles directly in the liquid matrix [33,34,35,36,37]. These methods are mainly based on elastically scattered light from the viral capsid [32,36,37,38]. In contrast to the bright-field microscopy, dark-field microscopy is a well-known technique for clear nanoparticle identification, through reduction of visual noise and allowance of the bright crystalline contrast, in order to differentiate the nanoparticles from the remainder of the environment [39]. The dark-field microscopy has the advantage of the detection of nanoparticles in complex media, including wastewaters or biological media [40]. 

Nanoparticle visualization via dark-field microscopy was demonstrated for gold nanoparticles based on their plasmonic spectra [41]. Total internal reflection dark-field microscopy (TIRDFM) was also demonstrated successfully for label-visualization of influenza viruses [42]. It is noteworthy that most attempts to visualize VNPs with dark-field microscopy rely on other labelling approaches, such as using quantum dots or gold nanoparticles, for real-time visualization [43]. These methods can track nanoparticles for longer timescales compared to fluorescence methods, however, they still have some limits. The limitation of gold nanoparticle concentration for detection and the noise introduced from background scattering, such as dust in the imaging chamber in most dark-field detection methods, poses a challenge for real-time detection [40]. Using dark-field microscopy to detect elastically-scattered light improves the detection sensitivity by reducing the background intensity [37]. 

**Figure 1:**
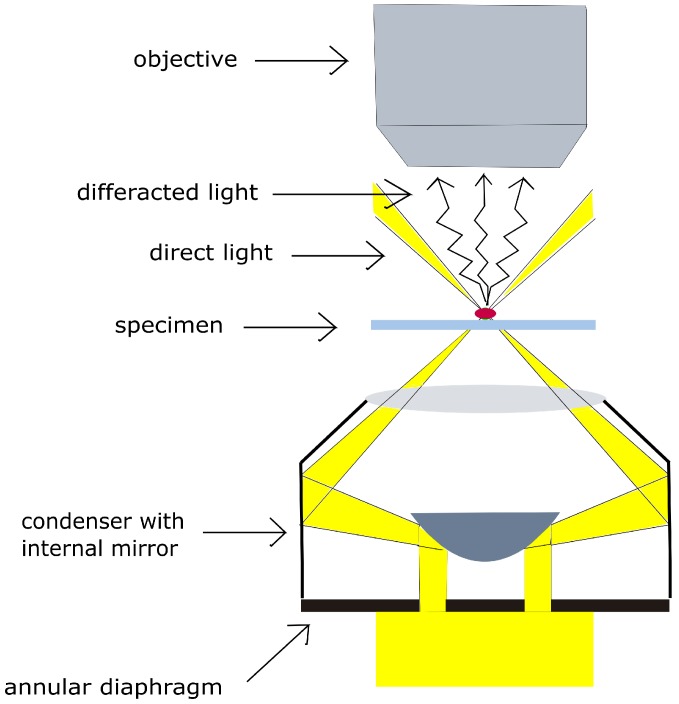
Scheme of nanoparticle illustration under CytoViva enhanced dark-field microscope with a hollow cone condenser. Only diffracted rays are collected by the objective.

In this work, we propose a new technique for label-free visualization and real-time, in situ imaging of VNPs based on enhanced dark-field optical microscopy. A CytoViva^®^ (Auburn, AL, USA) enhanced dark-field optical microscope was used to observe nanosized particles by detection of elastically-scattered light, without the use of any labels. The hollow cone of light in CytoViva allows no light to be transmitted through the microscope objective in the absence of a scattering source; therefore, only scattered light may be imaged (Figure 1). CytoViva’s patented dark-field condenser illuminates the sample more precisely than the standard microscope condenser by the focusing fixed-geometry, highly-collimated light at oblique angles on the sample, improving the signal-to-noise ratio up to seven times and enabling resolution beyond diffraction limits [38]. This study aims at visualizing and analysing the two-dimensional self-assembly of bacteriophage T4 using an enhanced dark-field optical microscope. Tracking of VNPs was performed through image analysis of videos recorded exclusively from the scattered light originating from the virus nanoparticles.

## 2. Materials and Methods 

### 2.1. Bacteriophage Propagation and Purification

*Escherichia coli* BL21 (5 mL each) was grown overnight in Luria broth (LB) at 37 °C. A 5 mL aliquot of this culture was added to 200 mL of sterilized LB and incubated under shaking for 3 h. Another 5 mL aliquot was inoculated with 100 µL of bacteriophage T4 stock (10^12^ PFU/mL (Plaque Forming Units/mL)), plaque forming units per mL) was incubated for 15 min at room temperature, and added to the first 3-h culture and incubated while shaking overnight at 37 °C. 

The phage suspension was purified based on PEG8000/NaCl aqueous two phase technique (ATPS) [44], and according to methods described in the literature [45]. Briefly, the overnight cultures were centrifuged at 6000× *g* RPM (RC-6, Sorvall, Thermo Scientific, Waltham, MA, USA) for 20 min, the supernatant was recovered and sterile-filtered. The supernatant was treated with nuclease solutions (final concentration of 1 μg/mL, 30 min at room temperature). The supernatant was subsequently mixed with 1 M NaCl and PEG 8000 (10 *v*/*w*%) and stirred overnight at 4 °C to precipitate phage particles. The precipitated phage were spun down by centrifugation (14,000× *g* RPM, 10 °C, for 3 h). Finally, the phage suspension was purified using ultracel-100k filters (Amicon Ultra Centrifugal Filters, Millipore, Carrigtwohill, Co., Cork, Ireland). The precipitated phages were re-suspended in salt medium (SM) buffer (containing 5.8 g/L NaCl, 120 mg/L MgSO_4_, 50 mL of 1 M Tris-HCl, pH 8) and sterile filtered. This method resulted in a phage titre of 10^10^–10^12^ PFU/mL. To determine the phage titre, ten-fold serial dilutions were prepared, and after mixing with overnight host bacteria the solutions were added to molten soft agar, which was spread over the plates. The plates were incubated overnight at 37 °C and the number of plaques were enumerated. To remove excess salt, bacteriophage suspension was dialyzed against distilled water for 24 h (Using regenerated cellulose membranes with MWCO: 14,000), followed by sterile filtration and phage titre count.

### 2.2. Experimental Setup: Enhanced Dark-Field Microscopy and Monitoring of Evaporation Process 

An optical Olympus BX51 polarizing microscope (10× UPlan, NA-0.3) equipped with an integrated dark-field illumination system (CytoViva^TM^, Auburn, AL, USA) was used to observe phage droplet evaporation on a borosilicate glass coverslip. The glass substrates were cleaned by acid washing and rinsing with ethanol 70% prior to the experiments and mounted in the microscope holder. Aqueous droplet of 0.5 µL containing T4 phage nanoparticles was gently pipetted and placed onto the clean glass coverslip. The droplet was left at room temperature, to evaporate until it dried completely. The drying procedure was monitored with 10×, 20×, and 40× microscope objectives and acquired as 1280 × 720 resolution videos, captured with a digital Carl Zeiss camera (12 MP, f/2.8, 1.77 µm per pixel size) that was mounted onto the ocular lens. The captured movies were analysed by image processing using MATLAB R2015a (MATLAB 8.5) software for tracking phage particles. 

### 2.3. Particle Tracking and Image Analysis

For particle tracking analysis, the captured movies were converted into still frames and each frame was processed using the mean-shift tracking algorithm. 

The tracking procedure was initiated by defining a local feature (i.e., the brightness level of a phage particle) around the object to be tracked. In the first frame, a small window was located around the particle. While the particle was changing its location through consecutive frames, the tracking algorithm shifted the window to keep its centre on the defined feature. 

To reinforce the tracking when particles were moving side by side to each other, a series of features, instead of one, could be taken into consideration. These features can vary from a simple pixel brightness, to more complex ones, such as the distribution of pixel normal vectors. In this work, a combination of two features, brightness level and elliptical diameters, insured that the particles were distinguishable from each other in situations where particles were too close to each other. The brightness level of each particle is obtained by averaging all the intensity values belonging to a particle phage. Note that since particles were captured as bright spots in all the frames, a static thresholding technique was applied to segment them from the dark background. Therefore, having segmented them, it was then possible to fit an ellipse to them in order to find approximated elliptical diameters.

The mean-shift methodology has been adopted from the code implemented by Bernhardt [46]. However, the code has been slightly modified in order to fulfil the requirements of the current research. The modifications included the reinforcement of particle features and the evaluation of particles trajectory.

The initial location of each phage particle was manually identified; the algorithm then automatically traced the location of that particle in subsequent frames. Conventional mean square displacement (MSD) was plotted against time lapses and the resulting curves were used to calculate the diffusion coefficient of phage particles in each suspension. Each analysis was performed on a dataset consisting of three to five particles (randomly selected) for each suspension. For each particle the analysis was carried out on 150–450 frames with time lapses of 0.2 s, 1 s, and 2 s (corresponding to time lags of *τ* = 50, 10, 5, respectively). Both tracking and MSD computation were performed using Mathworks MATLAB R2015a (MATLAB 8.5). Further information on data acquisition and MSD computation, available in the Supplementary File S, together with MATLAB code, are provided in the Supplementary Software File.

## 3. Results and Discussion

Assembling viruses on surfaces may lead to novel structures with high functionality in various areas, such as energy storage (as rechargeable batteries and solar cells), and bioengineering (as novel extracellular matrices and sensitive colour sensors) [47,48]. Drying induced assembly of viruses is a simple method to fabricate such functional structures. Gaining a fundamental understanding of the drying process and how it affects the assembly of viruses (and the final dried pattern) will affect our ability to control the self-assembly of VNPs. 

During the drying process, particles undergo two types of motion—fluid flow-driven transport and particle self-diffusion—both being space- and time-dependent [49]. Fluid flow transfers nanoparticles towards the free spaces, while particle self-diffusion decreases concentration gradients. These two parameters ultimately determine the shape of the self-assembled structure [50,51].

In this work, we used dark-field microscopy to visualize the drying process, and analysed the virus motion and fluid flow during drying of phage suspensions. Dark-field microscopy allowed monitoring of the phage nanoparticle motion in real-time, enabling the tracking of the nanoparticles motions without disturbance of the secondary agents (e.g., flurophores) and enabled real-time investigation of the drying dynamics. We tracked VNPs in movies captured using the microscope software under various conditions and analysed evaporation-driven fluid flow and evaporation-driven effects. 

Furthermore, the effects of salt and phage concentration on virus motion and fluid flow have been assessed by drying salt-free phage suspensions at low and high phage concentrations, as well as phages in physiological salt concentrations as an additional control.

### 3.1. Analysing the Bacteriophage Motion and Its Influence on Phage Assembly onto the Glass Coverslip

**Figure 2:**
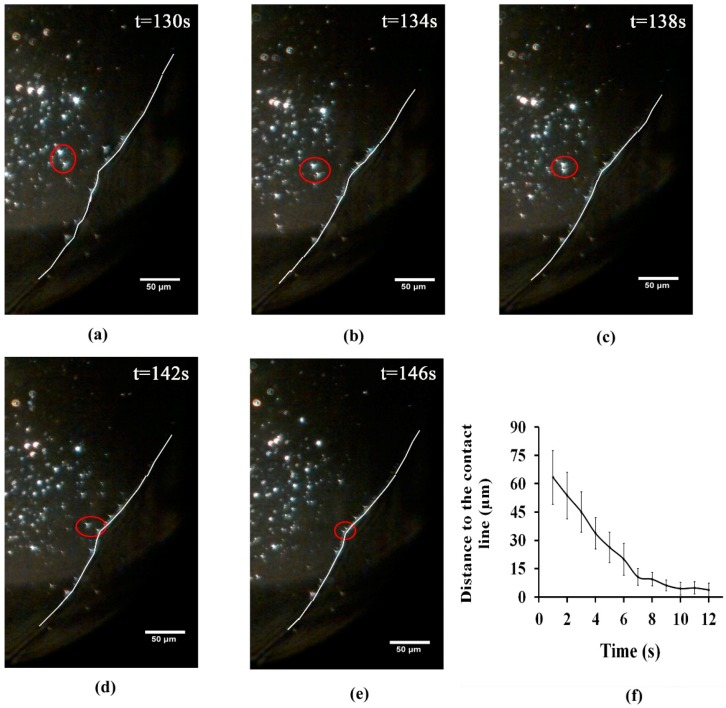
Sequential images (**a**–**e**) showing phage movement towards the contact line for diluted dialyzed phage (10^9^ pfu/mL); and (**f**) illustrates phage distance to the contact lines vs. time for the randomly-selected phage nanoparticles near the contact line, within 3–5 min after droplet contact with the glass coverslip.

Virus motion was assessed through quantification of phage nanoparticle diffusion coefficient in various suspensions. The diffusion of phage nanoparticle was quantified by monitoring particle trajectories during the drying process. It is known that the particle motion during the drying process accelerates towards the contact line. Hence, the diffusion coefficient was calculated through a tracking method, when the fluid flow is slow enough, and compared with the standard diffusion coefficient of phage T4 at the same temperature, as reported in the literature [52,53,54]. VNPs tracking was performed by processing captured movies obtained during evaporation procedure using a Mean-Shift algorithm of kernel-based object tracking. Figure 2a–e shows the image sequences of phage T4 movement (as motions of bright spots on a dark background) near the contact lines (the white line), while Figure 2f illustrates the distance of randomly selected phage nanoparticles (near the contact line) from the liquid-solid contact line vs. time, within 3–5 min after droplet contact with the glass coverslip. It should be noticed that the study on the phage diffusion behaviour in this study was limited to the conditions under slow fluid motion. This decreased the dynamics of VNPs motion and increased the quality of the signal obtained from individual VNPs, allowing for better visibility.

The dominating forces, responsible for transporting nanoparticles towards the contact line, are induced by the combination of capillary flow and diffusion mechanisms. Shen et al. [55], proposed two important time scales for the nanoparticles transportation near the contact line, in order to form a “coffee ring” structure. The first time scale is related to the diffusion-dominated transport mechanism of nanoparticles towards the contact line. The second time scale contributing towards the capillary flow mechanism [56,57] (induced by droplet evaporation at a pinned contact line), is related to the time for the two adjacent nanoparticles near the contact line to meet each other. By comparing these two time scales it was shown that the velocity of nanoparticle diffusion towards the contact line was several times higher than the velocity of nanoparticles movement induced by capillary flow [55]. Accordingly, Figure 2f shows that randomly-selected phage nanoparticles with various distances to the contact line (the vertical bars) are transported with higher velocities within the first 6 s time interval due to the diffusion-limited mechanism. However, their movements occurred at much lower velocities and in almost similar distances to the contact line within the next 6 s time interval, because the nanoparticle velocity influenced by the capillary flow is smaller than the diffusion velocity [55].

**Figure 3:**
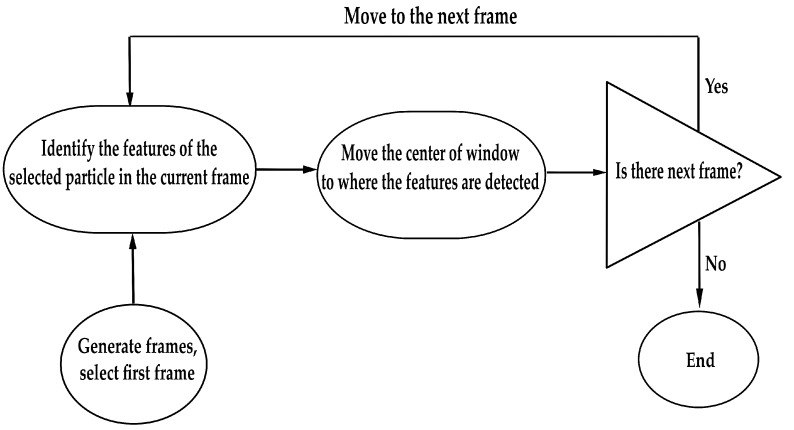
Flowchart describing the method for nanoparticle tracking using MATLAB software.

In order to find the trajectory of each moving object, assessing the location of the particle in each frame was required. For this purpose, the mean-shift algorithm was used to locate the most likely region where the particle moves. Particle properties, such as size and colour distribution, have been considered to strengthen the tracking. It was assumed that the colour intensity of a particle changes with regard to the 3D-Gaussian function. Therefore, mean-shift iteratively finds and refines the particle location considering the size and colour intensity profile in the particle. A flowchart detailing the algorithm developed for these experiments, using MATLAB software, is provided in Figure 3.

**Figure 4:**
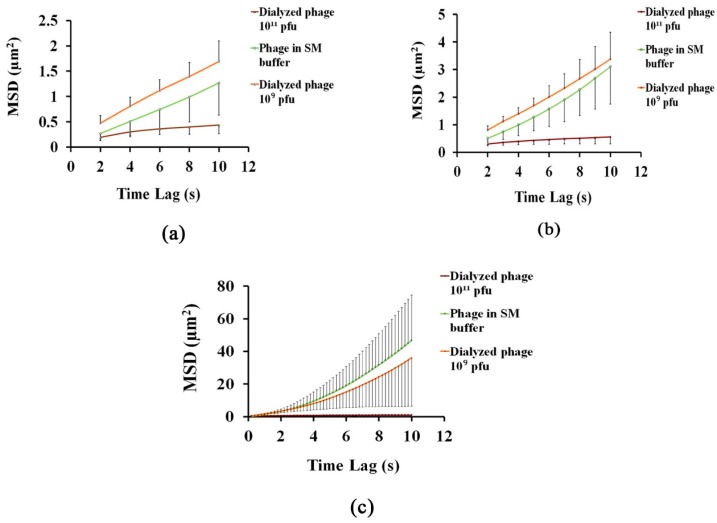
Mean square displacement of phage nanoparticles under different conditions with (**a**) *τ* = 5; (**b**) *τ* = 10; and (**c**) *τ* = 50.

Since the location of a particle is determined in each time frame, standard methods to calculate the mean-squared displacement (MSD) could be used to analyse the trajectory of each particle. MSD may be considered as the squared distances during a particle’s oscillation in the position over an identified time interval (with a certain length of Δt). Figure 4 shows the influences of salt ions and phage concentration on the mean-squared displacement of phage nanoparticles under conditions where the fluid motion is slow (as Movies S1 to S3). Time lags (*τ*) were determined as a function of a certain length of time interval Δt, and the number of time intervals n, (*τ_n_* = *n*Δt). In this study, time lags of *τ* = 5, 10, and 50 were considered to analyse the MSD values and, subsequently, the diffusion coefficient for phage nanoparticles in each solution. For the duration of the observation, increasing time lag, which will lead to increasing *n*, yields more time points for accurate estimation of displacement. However, the results were accompanied with a larger variance with increasing *n* (error bars in Figure 4c), because the uncertainty of the particles position over larger *n* values increases as the reciprocal of the square root of the displacements [46].

In order to analyse the diffusion coefficient of phage nanoparticles, the slope of the best fitted lines for each MSD curve, as the average diffusion coefficient, was obtained and compared with the standard diffusion coefficient of phage T4. The standard diffusion coefficient for *E. coli* bacteriophage T4 has been reported in the literature to be in the range of 4 × 10^−8^ to 8 × 10^−8^ cm^2^/s [52]; this value is highly dependent on phage concentration. However, it has been pointed out that the concentration of electrolyte has no significant effect on the diffusivity of phage T4 [53]. Table 1 presents the phage diffusion coefficients calculated for the various conditions used in this work.

Based on Table 1, the average diffusion coefficient of bacteriophages in SM buffer is within the range of standard diffusion coefficients for the same phage as reported in the literature. Hence, it can be interpreted that phage nanoparticles’ motion in SM buffer was dominated by random Brownian diffusion and, therefore, phage adsorption to the glass substrate is a diffusion-limited process [54]. However, deviation of phage diffusion coefficient from the standard value, for suspensions of diluted (10^9^ PFU/mL) and concentrated (10^11^ PFU/mL) phages, suggests that the possibility of phage adsorption to the substrate may influence the normal diffusive motion of nanoparticles, as well as on the final dried pattern. 

### 3.2. Analysing Fluid Flow through Monitoring Drying Process and Final Dried Patterns (Macroscopic Observations)

The movies captured with the dark-field microscope showed nanoparticles motions inside the droplet, upon placing a droplet containing phage nanoparticles in contact with glass coverslips. The appearance of salt crystals was evident upon drying of the non-dialyzed suspension (Supplementary Figure) but the dialyzed suspensions did not exhibit salt crystals, but rather exhibited patterns containing two main regions: boundary and inner part (Supplementary Figure).

**Figure 5:**
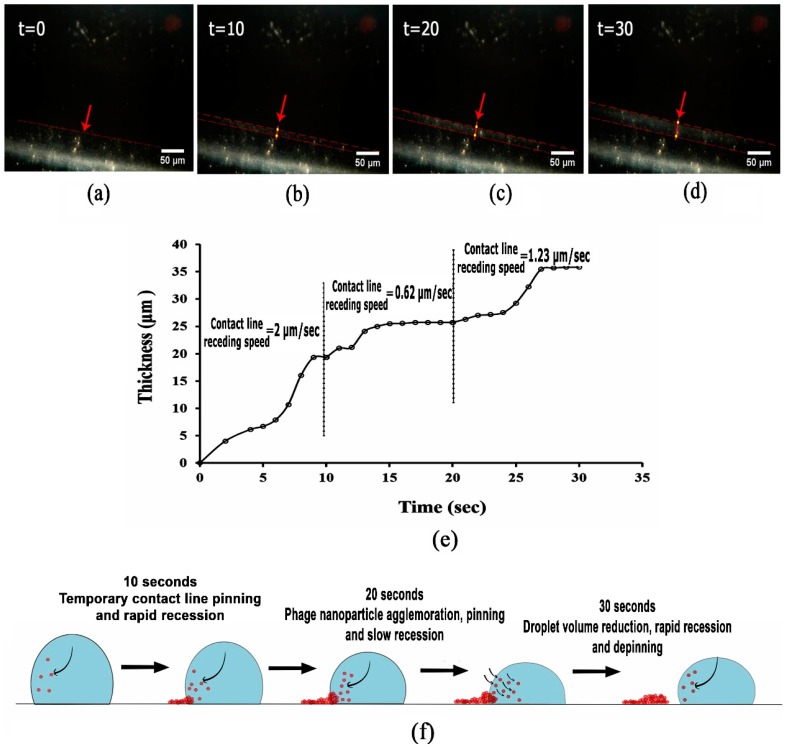
Sequential images derived from captured movies during drying of concentrated phage suspension, showing (**a**–**d**) concentric ring formation at 36% of drying process, during 30 s in 10 s intervals, using a 20× objective; (**e**) the contact line receding speed; and (**f**) a schematic diagram of the pinning-depinning process (the black arrows show the direction of phage nanoparticles motion towards the contact line during pinning and towards the centre of droplet during depinning processes).

The dynamics of phage nanoparticle motion in the fluid flow was apparent in the captured video from drying process of dialyzed solutions with high and low concentrations. In the first 15 min, for the high concentration phage suspensions, and 9 min, in the case of the diluted phage suspensions, the movement of the phage nanoparticle presented an outward motion, following pinning/de-pinning process and formation of repeated concentric rings (Movies S1 for higher phage concentrations and S5 for lower phage concentrations, Figure 5). The pinning/de-pinning process is an evaporation-driven effect and is the result of Stokes-Darcy transition (the two main physical laws governed the initial drying processes) [58,59], which was directly observed for both high concentration (Movie S1) and diluted phage samples (Movie S4) through the jumps of the contact line and the variation in the contact line position. The process has been explained by deposition of particles at the contact lines during pinning process, following by contact line jumps between pinning sites with droplet shrinkage, resulting in the formation of repeated concentric rings [60]. These observations confirm similar ones in a study on evaporation mechanism of rod-like viruses, reported in the literature [61]. Nevertheless, Moffat et al. [62], proved that the pinning step is not “absolute” and small contact line recession may occur during the pinning process.

Figure 5a–d shows the formation of concentric ring at 36% of drying process of the high concentration phage suspension after 30 s of drying. The variation of the receding contact line speed (slow or fast moving contact line) with variations in dried deposit from narrow ring to wide ring, has been discussed elsewhere through assessing the dynamics of contact line [62,63,64]. Moffat et al. [62], revealed that for the solutions with higher nanoparticle concentrations, the accumulation of nanoparticles near the contact line, as the result of advection during evaporation, induced the viscosity at the contact line, and subsequently the dynamic pinning behaviour. Figure 5e illustrates the average speed for the contact line receding of 2 µm/s for the first 10 s, 0.62 µm/s for the second 10 s, and 1.23 µm/s for the third 10 s.

As shown in Figure 5f, the higher receding speed of contact line during the first 10 s could be due to the high concentration of phage nanoparticles carried towards the contact lines at the droplet periphery (Movie S1 and arrows shown in Figure 5f), leading to the so-called temporary contact line pinning [62], passing from one nanoparticle to the next. The reduced speed of contact line recession, observed at the second 10 s period could be due to higher phage nanoparticle deposition, which prevents the contact line from receding and keeps the droplet at its contact area [63,65]. Increasing the contact line speed in the third 10 s, as the result of depinning process, is due to the continuous evaporation of the droplet at an almost constant contact area, followed by the reduction of the droplet volume, which can exceed the contact line recession towards the centre in order to reach a thermodynamic equilibrium state [66].

From the drying process movies, it was possible to observe and follow the complete drying process, for both diluted and concentrated phage suspensions. Upon the evaporation of the phage droplet, it was observed that the motion of phage nanoparticles in the concentrated suspensions occurred in a more confined area (Movie S1) than for the diluted suspensions, consisting of more free spaces (Movie S4), leaving behind several distinct concentric coffee rings. On the contrary, the patterns formed after drying of phage nanoparticle suspension with low concentrations (Supplementary Figure), contained no distinct bands (coffee rings), as opposed to what could be observed for high phage concentration.

**Figure 6:**
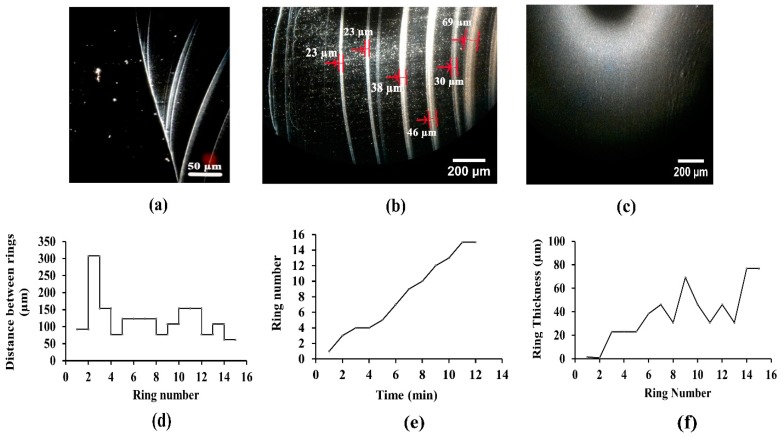
Patterns after drying of phage (dialyzed) with higher phage concentration (10^11^ PFU/mL). (**a**,**b**) Multiple coffee rings and (**c**) inner dried regions. The coffee rings thicknesses are pointed by arrows in (**b**). The characteristics of coffee rings formed for the concentrated dialyzed phage suspensions are shown as (**d**) the distance between rings vs. the ring number; (**e**) the ring number vs. time; and (**f**) the ring thickness vs. ring number.

Figure 6 shows images of drying stages and the characteristic plots of multiple-ring formation, for concentrated phage suspension. For the solutions with high phage concentrations, increasing phage nanoparticle concentration at the contact lines may result in the formation of a gel-like agglomeration in liquid-solid contact edges and, consequently, result in slow movement of gel-like phage agglomerates towards the centre of the droplet, during the de-pinning process (Movie S1). The solvent evaporation from such gel-like assemblies during their movement may induce the formation of highly dense inner contact regions [67].

Such behaviour was observed as the evaporative deposition of phage nanoparticles in the distinct contact rings (arrows in Figure 6b), and 15 pinned contact rings were visualized from the drying process movies, during the initial 13 min of evaporation. The observation showed that the contact rings were accumulated in one side of a droplet, while their ends joined together (Figure 6a). It seems that the pinning-depinning process occurred on one side of the droplet edge, whereas the other side was pinned permanently, which is in contrast with most of the studies, expressing the pinning-depinning process symmetrically, during evaporation of droplets with spherically-symmetrical configurations. Nevertheless, Sáenz et al. [68], implied that the droplet geometry dictates the particle deposition and evaporative dynamics, in a way that a non-uniform coffee ring effect could be the result of non-uniform evaporation flux along the non-spherical droplet interface. The evaporation flux is larger at the corners than along the rest of the perimeter, leading to a preferential particle accumulation in these regions. Hence, such observations in this study may be due to the unintentional spreading of droplets with non-spherical geometry. 

Figure 6d–f illustrates the coffee ring characteristics for the concentrated phage suspension (without salt). As the droplet shrinks continuously, the thickness of the contact rings increases due to the higher phage concentration at the centre and, consequently, the spacing between rings decreases. 

Phage nanoparticles in the low-concentration suspension probably had enough free space to diffuse and recirculate towards the contact lines and backwards, within the droplet (Movie S2). Therefore, at the very initial stages of drying, they are arranged into closely-packed particles in the contact lines as the result of both fluid flow and particle diffusive motion (Movie S4). Moreover, Kajiya et al. [69], proved that the concentration of particles near the contact line is quite large for the suspensions with both high and low colloid concentrations, which was due to the outward flow and was visible at the early moments of the drying process. As evaporation progressed, the flow velocity towards the contact line increased in order to replenish the evaporated liquid, while the droplet height was vanishing [70]. The decrease in liquid surface area transported the trapped diffusing particles from the air-liquid interface, towards the substrate surface. Increasing the particles’ density near the surface decreased their free motion and resulted in their deposition in thicker contact areas rather than the previously-observed thinner ring-like structures previously observed (Supplementary Figure) [71]. Hence, the dried patterns of phage nanoparticles at the edges are not in the form of repeated contact rings (Supplementary Figure), as it was the case for the edge patterns observed for the concentrated dialyzed phage (Figure 6a–c).

The inner structures of the dried patterns of both diluted and concentrated phage suspensions are quite different, representing an irregular disordered structures (Supplementary Figure) and a uniform pattern (Figure 6d), respectively. The disordered irregular inner structures of dried patterns for diluted phage suspension may be due to the non-uniform distribution and the increasing velocity of phage nanoparticles as the result of air invasion (entering of air to the droplet) [72,73] at the final drying stage. Visualization of the final stage of evaporation, for suspensions with lower phage concentration, showed that the phage nanoparticles were entrapped as small or large clusters in the bulk flow and transported in the direction of air invasion (Movie S5). Therefore, phage nanoparticles were packed into a non-uniformed structure in the central part of the patterns. The non-uniform structure of the suspensions at the final stage of drying has also been observed by other authors [72,74]. On the contrary, in the concentrated suspension, due to the confined diffusive motion of nanoparticles, phages were prone to be trapped by the surface and compacted at the centre of droplet/substrate interface since the initial stages of evaporation. Therefore, the formation of packed nanoparticles caused the nucleation of capillaries at the centre of the droplets [75]. Consequently, the thin film of liquid at the air-liquid interface in the final stage of drying was disrupted, as single capillary nucleation occurred inside the droplet and, therefore, the solution shrank from different directions (Movie S6).

**Figure 7:**
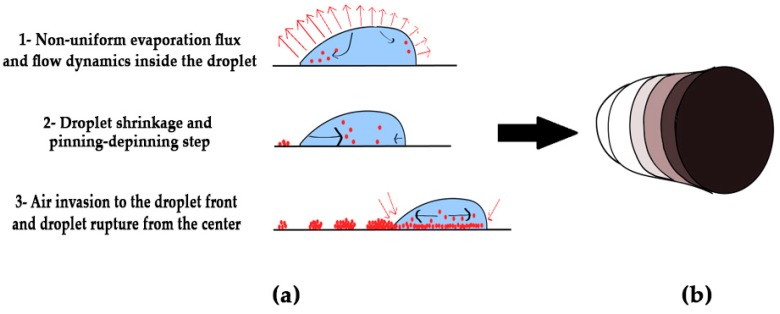
The schematic diagram of the drying process for concentrated dialyzed phage suspension, (**a**) the drying steps of droplet including: evaporation (formation of coffee rings and dense regions), and air invasion (formation of central area); and (**b**) final dried patterns. The colour gradient represents different nanoparticle densities deposited onto the substrate during the drying stages.

Figure 7 illustrates a schematic diagram of the drying process for the high-concentration phage suspension, according to the observations presented in Figure 6. Briefly, the phage nanoparticles deposited at the contact lines at the early stage of drying, due to the highest evaporation flux at the contact regions of the droplet. Increasing the non-uniform evaporation flux along the droplet surface, enhanced the flow dynamic and radial velocity at greater distance from the droplet centre. Hence, the accumulation of nanoparticles at the droplet-substrate interfaces, may sediment and pin the contact lines. At later time points of the evaporation, the bulk drop volume decreased, causing the nanoparticles to flow toward the drop centre and de-pinning of the contact lines. The final seconds of evaporation occurred along with the air invasion to the droplet front, while the thin film of the liquid at the air-liquid interface was ruptured from the centre. The colour gradient in Figure 7b represents the different phage densities deposited onto the glass coverslip during the evaporation process. Phage deposition was observed to increase from the initial coffee rings towards the dense inner contact lines and inner area.

Finally, correlating the diffusion behaviour of phage nanoparticles from tracking results (Figure 4) to the images obtained after drying of phage suspensions (Figure 6 and Supplementary Figure) confirmed that deviation from normal diffusive motion, as the result of phage nanoparticle entrapment at the surface, influencing the evaporation process and the dried patterns features. 

Boulogne et al. [51] stated that the drying of colloidal suspension is a competition between the fluid flow causing the particles’ accumulation at the free surface, and the diffusive motion which smooths the concentration gradient. The formation of a skin at the surface of a drying film (as the result of fluid flow), or the consolidation in the bulk phase (due to the diffusive motion), can be distinguished through measuring the Peclet number. Hence, in future works, diffusion coefficient, film thickness, and the evaporation rate could be assessed in order to obtain the Peclet number as an index to distinguish between skin formation and consolidation in the bulk. 

## 4. Conclusions

Bacteriophage immobilization onto functional materials through evaporation is a simple, fast, and effective method to develop a suitable bioactive material. Dark-field enhanced optical microscopy (CytoViva) was applied in this research to visualize and track phage nanoparticle movements in an aqueous liquid and observe their final assembly patterns after evaporation. 

Phage arrangement on the surface originated from a competition between fluid flow and nanoparticle motion during evaporation. Assessing the fluid flow and phage motion during the drying process of aqueous solutions of phage suspension with different concentrations clarified the impact of parameters, such as liquid salinity and phage concentration, on the drying process and final dried patterns. It was found that thin distinguishable rings and a uniform inner pattern were formed at higher concentrations, while thicker contact regions at the edges, as well as a disordered inner pattern have been observed for the lower concentrations. 

Aside from the parameters investigated in this approach, there are still several other factors, such as pH, surface type and hydrophobicity, temperature, and different salt type and concentrations that require further research to reach a thorough analysis of phage immobilization onto the surfaces.

Tracking of phage nanoparticles is an advanced capability of the technique proposed in this study, which provides insights for inferring the phage-based structures under various conditions and which may be regulated. Calculation of the phage nanoparticle diffusion coefficient through the tracking method and its comparison with the standard diffusion coefficient at the same temperature, may lead to defining the phage adsorption process onto a substrate. A diffusion-limited adsorption can be attributed to a system with normal phage diffusive motion, while there is less phage entrapment by the substrate during evaporation. However, the effect of concentration on the diffusive motion of phage nanoparticles could result in the possibility of phage adsorption to the substrate, as well as on the final dried pattern. 

The label-free technique introduced by this paper is a useful method for the in-situ study of both monitoring of the microscopic particles and the in-situ study of the self-assembly process. This technique has the advantage of being simple, without any laborious preparations, for repeatedly applying to track VNPs. Therefore, it can be extensively used for a range of practical applications based on phage templating. However, the results are still in the very early stage, therefore requiring further work to reach the full capacity of this technique.

Our tracking and self-assembly results provide new insights for tuning the self-assembly behaviour of viruses by manipulating the experimental conditions in order to fabricate the desired architectures. Comparing viral and non-viral motion in a simulated designed environment to that of biological ones with the presented method, may help with the studies of gene and drug delivery for future works.

T4 Bacteriophage immobilization on substrates is widely investigated for biosensor applications, as well as the formation of novel bioactive surfaces used for the prevention of biofilm formation in medical applications. Patterned immobilization of T4 phages on top of surfaces may lead to better capture of pathogens in different applications. 

## Figures and Tables

**Table 1:** Diffusion coefficient of phage T4 suspensions used in this study.

T4 Phage Suspensions	Diffusion Coefficient (cm^2^/s)
T4 phage suspension in water at 23 °C (10^8^ to 10^9^ pfu/mL) [52]	4 × 10^−8^–8 × 10^−8^
T4 phage suspension in SM buffer (10^9^ pfu/mL)	4.8 × 10^−8^
Concentrated T4 phage suspension in water (10^11^ pfu/mL)	5.6 × 10^−10^
Diluted T4 phage suspension in water (10^9^ pfu/mL)	3.6 × 10^−8^

## Supplementary Materials

The following are available online at http://www.mdpi.com/2079-4991/8/1/1/s1, Figure S. Final dried patterns of (a–c) phage suspensions containing salt ions (phage in SM buffer); (d–f) dialyzed suspensions with high phage concentration (10^11^ PFU/mL); and (g–i) dialyzed suspensions with low phage concentration (10^9^ PFU/mL). Supplementary Software Files: Supplementary File S. Information on data acquisition and MSD computation. MATLAB Code: Software codes for tracking nanoparticles; Movie S1. Phage nanoparticle motion in the concentrated dialyzed phage suspension (10^11^ PFU/mL). Movie S2. Phage nanoparticle motion in the diluted dialyzed phage suspension (10^9^ PFU/mL). Movie S3. Phage nanoparticle motion in SM buffer (10^9^ PFU/mL). Movie S4. Phage nanoparticle motion towards the contact line in the diluted dialyzed phage suspension (10^9^ PFU/mL). Movie S5. Final drying stage of the diluted dialyzed phage suspension (10^9^ PFU/mL). Movie S6. Final drying stage of the concentrated dialyzed phage suspension (10^11^ PFU/mL). Movie S7. Tracking procedure using the mean-shift algorithm equipped with a kernel function.
