# Supplementary material for: A New Label-Free Technique for Analysing Evaporation Induced Self-Assembly of Viral Nanoparticles Based on Enhanced Dark-Field Optical Imaging
Source: Nanomaterials (Basel). 2017 Dec 22;8(1):1. doi: 10.3390/nano8010001 (PMC5791088; doi:10.3390/nano8010001)
Supplement: Supplementary file 1 [file nanomaterials-08-00001-s001.zip › nanomaterials-239995-supplementary.docx]

A New Label-Free Technique for Analysing Evaporation Induced Self-Assembly of Viral Nanoparticles Based on Enhanced Dark-Field
Optical Imaging

Ima Ghaeli ^1,2,3,^*, Zeinab Hosseinidoust ^4^, Hooshiar Zolfagharnasab ^5^ and Fernando Jorge Monteiro ^1,2,3,^*


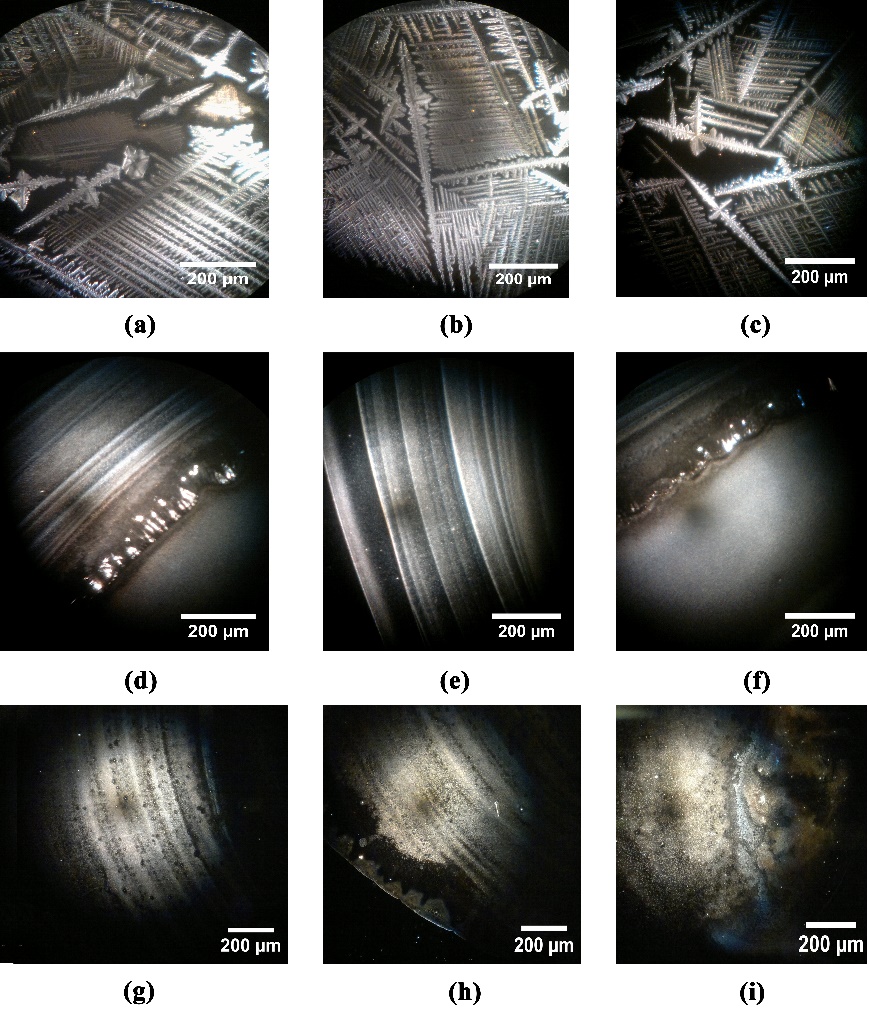


**Figure S:** Final dried patterns of (a-c) Phage suspensions containing salt ions (phage in SM buffer), (d-f) Dialyzed suspensions with high phage concentration (10^11^ PFU/mL), (g-i) Dialyzed suspensions with low phage concentration (10^9^ PFU/mL)
